# Automated insulin delivery during the first six months postpartum: pre-specified AiDAPT extension study

**DOI:** 10.1016/S2213-8587(24)00340-1

**Published:** 2025-01-27

**Authors:** Tara T.M. Lee, Corinne Collett, Simon Bergford, Sara Hartnell, Eleanor M. Scott, Robert S. Lindsay, Katharine F. Hunt, David R. McCance, Rebecca M. Reynolds, Malgorzata E. Wilinska, Judy Sibayan, Craig Kollman, Roman Hovorka, Helen R. Murphy

**Affiliations:** 1 Norwich Medical School, University of East Anglia, Norwich, UK; 2 Norfolk and Norwich NHS Foundation Trust, Diabetes and Antenatal Care, Norwich, UK; 3 Norwich Clinical Trials Unit, Norwich Medical School, University of East Anglia, Norwich, UK; 4 Jaeb Center for Health Research, Tampa, Florida, USA; 5 Cambridge University Hospitals NHS Foundation Trust, Cambridge, UK; 6 Leeds Institute of Cardiovascular and Metabolic Medicine, University of Leeds, Leeds, UK; 7 Institute of Cardiovascular and Medical Sciences, University of Glasgow, Glasgow, UK; 8 King’s College Hospital NHS Foundation Trust, London, UK; 9 Regional Centre for Endocrinology and Diabetes, Royal Victoria Hospital, Belfast, UK; 10 Centre for Cardiovascular Science, University of Edinburgh, Edinburgh, UK; 11 Wellcome-MRC Institute of Metabolic Science, University of Cambridge, Cambridge, UK

## Abstract

**Background:**

Clinical guidelines in the UK and elsewhere do not specifically address hybrid closed-loop (HCL) use in the postpartum period when the demands of caring for a newborn are paramount. Our aim was to evaluate the safety and efficacy of HCL use during the first six months postpartum.

**Methods:**

In an extension to a multicentre, randomised controlled trial, pregnant women with type 1 diabetes at nine UK sites were followed for six months postpartum. Eligible participants continued their randomly assigned treatment: standard insulin therapy with continuous glucose monitoring (CGM) or HCL therapy (CamAPS FX system). They were randomised on a 1:1 basis with stratification by clinical site using randomly permuted blocks. Primary outcome was the between-group difference in percentage time in range; TIR 3.9–10.0mmol/L (70-180mg/dL), examined during 0 to <3 months, 3 to 6 months, and over 6 months postpartum. Clinical Trial Registration: ISRCTN56898625

**Findings:**

57 participants (mean ± SD) age 31 ± 4years and pre-pregnancy HbA_1c_ 59.4 ± 10.5mmol/mol (7.6 ± 1.0%) were included. Mean time with glucose levels within target range was higher; 72 ± 12% vs. 54 ± 17% (adjusted treatment difference, 15 percentage points [95% CI, 7-22]) over six months postpartum. Results for hyperglycaemia (>10.0 mmol/L) and mean CGM glucose also favoured HCL (-14% [-23%, -6%] and -1.3mmol/L [-2.3, -0.3], respectively). Hypoglycaemia rates were low with no between-group differences (2.4% vs 2.6%). There were no treatment effect changes depending on postpartum period (0-<3 vs 3-6 months) and no unanticipated safety problems.

**Interpretation:**

HCL users maintained 70% time in range during the first six months postpartum, supporting continued use of HCL rather than standard insulin therapy.

**Funding:**

National Institute for Health Research, Juvenile Diabetes Research Foundation, and Diabetes Research & Wellness Foundation. Continuous glucose monitoring (CGM) devices were provided by Dexcom at a discounted price.

The daily management of glucose levels in type 1 diabetes (T1D) is challenging. Maintaining safe maternal glycaemia in the postpartum period is complicated by the profound physiological changes that occur after delivery and lifestyle changes associated with caring for a newborn. Following delivery of the placenta, insulin sensitivity dramatically increases, however, there is considerable inter-individual variability with some individuals requiring minimal exogenous insulin in the initial 12-24hrs^1,2^. During the months following delivery, changing maternal hormones, unpredictable daily routine, and variable maternal and infant feeding patterns further complicate diabetes management and insulin dose adjustment^3,2,4,5^. Sleep deprivation and exhaustion exacerbate the mental burden of glycaemic self-management alongside caring for a newborn – both of which demand constant attention. These postnatal diabetes challenges are further compounded by a gap in care as women transition from intensive antenatal support (2 - 4 weekly appointments) to general diabetes services (2 – 4 appointments per year) with many describing “being lost” and “not knowing who to turn to when they had trouble or needed support”^6^.

Hybrid closed-loop (HCL) systems are increasingly effectively used in T1D management across many populations (adult, paediatric, pregnancy) to help users meet glycaemic targets and reduce the mental burden of diabetes self-management^7–11^. In December 2023, the UK National Institute for Health and Care Excellence (NICE) updated its guidance for diabetes technology, supporting HCL use. Based on data from AiDAPT trial (Automated insulin Delivery Amongst Pregnant women with Type 1 diabetes, ISRCTN56898625), the NICE Technology Appraisal [TA943] now recommends offering HCL therapy use before and during pregnancy^12^. However, because postnatal studies were limited in size and scope, NICE did not specifically address the use of diabetes technology and HCL therapy during the postpartum period. As more women use HCL before and during T1D pregnancy, this omission and gap in postpartum diabetes management requires urgent attention, so that women are empowered to choose evidence-based therapy during this challenging period.

Four small studies have examined postnatal use of commercially available HCL systems. In the two UK Closed-Loop in Pregnancy (CLIP-03, CLIP-04) studies and American Pregnancy Intervention with a Closed-Loop System (PICLS) study, HCL therapy was initiated during pregnancy and continued postnatally. In the Canadian Closed-Loop Insulin in Mothers with Type 1 Diabetes and Baby feeding practices (CLIMB) study, HCL therapy was started denovo one week after birth. The UK CLIP studies reported safe inpatient use of previous prototype versions of the CamAPS FX system in 27 participants throughout labour, birth and the initial 48-hours postpartum^13^. We subsequently described target glycaemic attainment (77% time in range, TIR 3.9-10.0mmol/L, 70-180mg/dL) with low rates of hypoglycaemia (2.4%) among 12 participants who continued using CamAPS FX for six weeks postpartum^14^. The Canadian CLIMB study of 18 participants and American PICLS study of 23 participants reported similar glycaemic outcomes (CLIMB TIR 79% vs 78% HCL vs sensor-augmented pump and PICLS 75% vs 77% respectively) associated with use of the Medtronic MiniMed 670G and 770G HCL systems^15,16^. The CLIMB and PICLS studies also reported low rates of maternal hypoglycaemia (2% vs 6% and 5% vs 9% respectively). Whilst reassuring from a safety perspective, these feasibility studies do not demonstrate definitive proof of efficacy. They also included participants with near optimal glycaemia which limits their generalisability, in real-world settings.

In this study, we examined the continued use of CamAPS FX HCL therapy from pregnancy and its effects on maternal glycaemia from day one after delivery through the first six-months postpartum^17^. We also examined whether there were any changes in treatment effect during the earlier (0 to <3 months) or later (3 to 6 months) postpartum period.

## Methods

### Study design

This study was performed as an extension to AiDAPT (Automated insulin Delivery Amongst Pregnant women with Type 1 diabetes, ISRCTN56898625), a multicentre, parallel group, randomised controlled trial, recruiting pregnant women with T1D across nine UK National Health System (NHS) sites. Participants were randomly assigned during pregnancy to receive HCL (CamAPS FX system; intervention group) or to continue standard insulin therapy (multiple daily injections or insulin pump, and continuous glucose monitoring [CGM]; standard care group). The AiDAPT study protocol and primary results were previously published and are briefly summarised below^10,17^.

After pregnancy, trial participants were provided with three to four sensors allowing for up to six to eight weeks of postpartum CGM use to allow safe transition back to usual clinical care. However, approximately halfway through trial recruitment, the UK NICE Diabetes Pregnancy guidelines were updated to recommend 12 months of real-time CGM use for all pregnant women with T1D^18,19^. This was accompanied by ring-fenced pregnancy-specific funding to accelerate nationwide implementation of CGM use during 2021^20^. Thus, pregnant women with T1D who did not participate in the AiDAPT trial were allocated five to six months of NHS funded postnatal CGM sensors (i.e. based on women starting CGM at 10-12 weeks’ gestation and delivering around 36-38 weeks’ gestation). This raised an ethical dilemma, potentially disadvantaging AiDAPT trial participants who at that time point could only access six to eight weeks of postnatal CGM use.

Additional changes in maternity and diabetes service provision during and after the COVID-19 pandemic included increased clinical pressures among trial staff and restricted face-to-face appointments. As a result of the expanded access to CGM and service provision pressures, we sought Research Ethics Committee approval to extend the use of CGM sensors, with or without HCL therapy, to eligible trial participants for six months postpartum. This was approved (AiDAPT protocol version 5.0) to comply with national CGM recommendations and ensure safe postnatal transition for the remaining AiDAPT participants. Registration of the postpartum extension study was included in the AiDAPT trial registration (ISRCTN56898625) and was previously described in the published study protocol paper^17^.

### Trial participants

Pregnant women aged 18 to 45 years of age, with at least one year’s duration of type 1 diabetes and a HbA1c of 48 to ≤86 mmol/mol (6.5 to ≤10.0%) were recruited to AiDAPT prior to 14 weeks’ gestation. Those recruited after implementation of the postpartum protocol amendment approval (12^th^ November 2021) or those still pregnant or within six months of delivery at the time of amendment implementation and still using CGM or HCL therapy from pregnancy, were eligible for inclusion in postpartum follow up extension study.

### Randomisation and Masking

Participants continued their assigned treatments following randomisation during early pregnancy (median ~11 weeks’ gestation). They were randomised on a 1:1 basis with stratification by clinical site using a computer-generated randomisation system with randomly permuted blocks sizes of 2 and 4. Once a participant was randomised, both the investigator and participant were aware of the treatment assignment. Investigators were masked to the results until the study was completed. The primary outcome was based on the downloaded CGM data. Statisticians at the coordinating centre (Jaeb Center for Health Research) who used the CGM data to calculate the time in range were not masked to the study treatment.

### Trial procedures

Eligible participants at the time of implementation of the postpartum protocol amendment (those still pregnant or within 6 months of delivery and for whom CGM data were available) were approached. Those recruited after implementation of the postpartum amendment were consented at the same time as recruitment to the AiDAPT trial. Following delivery of their baby, participants in both groups received usual clinical care.

### Treatments

#### HCL Group

The HCL system used was as per the AIDAPT trial i.e. the CamAPS FX application (version 0.3.71, CamDiab, Cambridge, UK), hosted on an android smartphone (Samsung Galaxy S8-S12, Samsung, Suwon-si, South Korea). A Dana Diabecare RS insulin pump (Sooil, Seoul, South Korea) and Dexcom G6 CGM (Dexcom, San Diego, USA) communicated by Bluetooth with the algorithm for insulin administration and glucose monitoring respectively. Postnatal plans with starting guidance for pump and HCL settings were agreed between the woman and diabetes antenatal teams and documented prior to delivery, around 36 weeks’ gestation. Women were advised to switch to recommended starting postpartum settings immediately before caesarean section or as soon as the placenta delivered. Recommended initial postpartum settings included a personal glucose target of 6.0mmol/L (108mg/dL) and insulin to carbohydrate ratios of between 1:12g and 1:15g depending on infant feeding status. Following delivery, whilst still in hospital, participants titrated their own personal glucose targets, insulin-to-carbohydrate ratios (ICR) and pre-meal insulin boluses aiming for CGM TIR targets (70% time between 3.9-10.0mmol/L and <5% time below 3.9mmol/L). Women were also encouraged to use the “Boost” and/or “Ease off” features for at least 2-4 hours at a time if they felt that other setting changes were not fast enough to counter higher or lower glucose levels. They were encouraged to continue self-titrating their settings as needed when discharged from hospital with contact details for their usual NHS diabetes clinical support (adult diabetes services including diabetes specialist nurses and midwives), given the variable nature of insulin dosing and requirements between individuals and from day-to-day^21^.

#### Standard Care Group

Participants assigned to the standard care group continued their usual insulin therapy, multiple daily injections or insulin pump therapy, with clinical support from their local teams. During the postpartum period, insulin doses (both pre-meal insulin boluses and basal insulin doses) were titrated to achieve CGM targets (70% time between 3.9-10.0mmol/L, 70-180mg/dL).

#### Postpartum visits

Participants were followed up by telephone at 8-12 and 24 weeks after delivery, at which CGM data, insulin doses and delivery method, safety outcomes, and infant feeding status were reviewed. Participants received review of their diabetes management with adjustments to insulin dosing and advice if required. To assess diabetes and treatment-related lived experience, participants were sent open ended questions (Supplementary Materials page 2) to provide self-reported free text feedback on their lived experiences through descriptive writing.

### Outcomes

Primary outcome was the between-group difference in percentage of time with CGM glucose measurements in the postpartum target range; TIR 3.9-10.0mmol/L (70-180mg/dL). The six-month postpartum extension was split into two periods: from the day of delivery to three months and from three to six months post-delivery. In each three-month period, CGM outcomes were calculated overall and by time of day (day: 7am-11pm, night: 11pm-7am). Pre-specified secondary outcomes were percentage of time spent hyperglycaemic (>10.0mmol/L, > 180mg/dL) and hypoglycaemic (<3.9mol/L, <70mg/dL) and other sensor glucose metrics (mean CGM glucose and glucose variability metrics). Safety outcomes were severe hypoglycaemia, diabetic ketoacidosis and device-related adverse events.

### Statistical Analyses

A minimum of 300 hours of CGM data in each three-month period were required to calculate overall CGM outcomes, and a minimum of 200 and 100 hours of CGM data were required to calculate daytime and overnight CGM outcomes, respectively.

A repeated measures linear regression model was fit for the three-month period outcomes, with CGM outcome as the dependent variable adjusting for CGM outcome for pre-pregnancy insulin delivery method (insulin pump or multiple daily injections), CGM metrics during the baseline pre-randomisation run-in period (approximately 10-11 weeks’ gestation), and clinical site as a random effect. A point estimate and 95% confidence interval were calculated for the adjusted treatment difference based on the linear regression model. A two-sided p-value was calculated for the treatment effect based on the linear regression model, and a 5% level was used to declare statistical significance. Residual values were examined for an approximate normal distribution and homogenous variance. A histogram and q-q plot of the residuals were examined for an approximate normal distribution, and a residual vs fitted plot was examined for homogenous variance. If values were highly skewed, the model used a t distribution with 10 degrees of freedom for the errors. The same model was repeated with an interaction between postpartum period (0 to <3 months, and 3 to 6 months) and treatment group to examine if the treatment effect changed depending on postpartum period.

There was no imputation for missing data. The false discovery rate was controlled using the adaptive Benjamini-Hochberg procedure for multiple comparisons. Analyses were performed with the use of SAS, version 9.4. Qualitative software (Qualcoder version 3.5) was used to facilitate data coding and retrieval for the qualitative analysis of the lived experience data.

### Role of the funding source

TTML, SB, JS, CK and HRM had access to the raw data. Funders had no role in the design of the study; in the collection, handling, analysis or interpretation of data; or in the decision to submit the protocol manuscript for publication.

## Results

**Figure 1:**
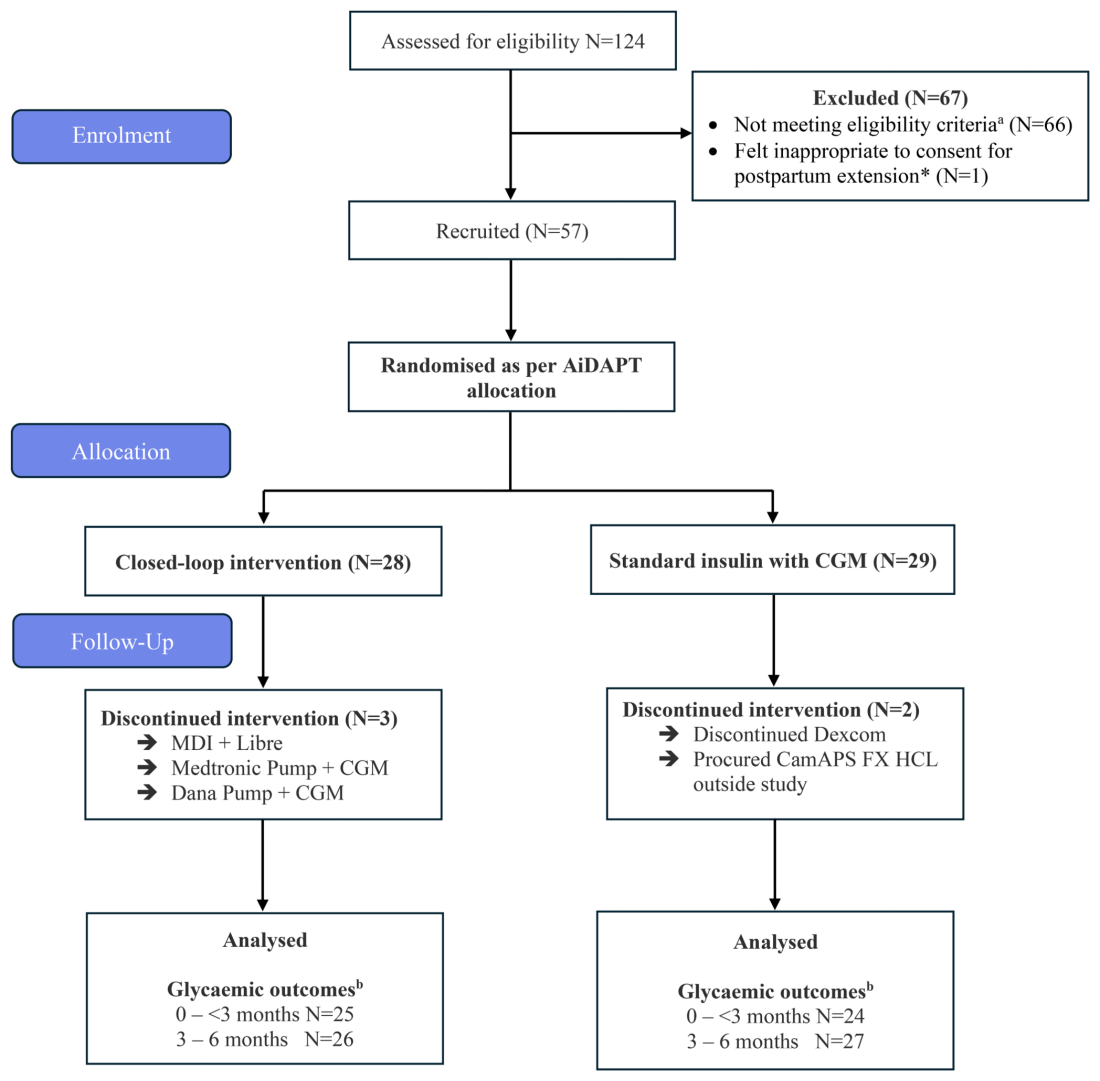
Consort Diagram a Reasons for not meeting trial eligibility criteria (N=66) were: Participants more than 6 months postpartum (N=60) Completed the study and returned to NHS care prior to extension being implemented and so these participants were no longer using hybrid closed-loop (CamAPS FX) or continuous glucose monitoring (Dexcom G6). (N=6) *This participant had a neonatal death and it was felt by both trial team and local site investigators inappropriate to approach to continue into postnatal extension. d In the HCL group, three participants had missing data in the 0-<3 months period and two had missing data in the 3-6 months period as assessed by continuous glucose monitoring. In the standard care group, five participants had missing data in the 0-<3 months period and two had missing data in the 3-6 months period as assessed by continuous glucose monitoring.

Out of the entire 124 AiDAPT trial participants, 66 were not eligible for inclusion in the postpartum extension study. Sixty participants (89.5%) were ineligible because they were already over 6 months postpartum when ethical and regulatory approvals were implemented at trial sites. Six participants (8.9%) were ineligible because they had already discontinued either HCL or control interventions (standard insulin with CGM) within the first 6 months. One control arm participant had a neonatal death and we deemed it inappropriate to approach her about participating in the postpartum study (Figure 1).

57 participants consented to continue their randomised allocation (standard care, which included CGM with multiple daily injections or insulin pump therapy vs HCL therapy) into the postpartum extension study. Postnatal participants were recruited from nine NHS clinical sites, spanning England, Scotland and Northern Ireland, had a mean age of 31 ± 4years and early pregnancy HbA1c 59.4 ± 10.5mmol/mol (7.6 ± 1.0%) (Table 1). Both groups spent ~70% TIR (3.9-10.0mmol/mol) at baseline (pre-randomisation) during early pregnancy (Table 2). Participant characteristics appeared similar for participants who consented to continue in the postpartum extension and those who did not (Supplementary Table 1).

In the standard care group, there were more participants for whom this was their first pregnancy, lower maternal BMI, higher gestational weight gain and more primary caesarean section deliveries. During pregnancy, AiDAPT participants in the HCL group spent more time in the pregnancy-specific target range of 3.5-7.8mmol/L (63-140mg/dL) from 16 weeks’ gestation until delivery^10^.

Five participants did not adhere to their randomised treatment allocation during the six-month postpartum extension period. Three intervention group participants discontinued CamAPS FX HCL use; one resumed her pre-pregnancy Medtronic insulin pump; one had increased personal/social difficulties and decided to discontinue HCL therapy and one resumed multiple daily injections at three months postpartum after multiple Dana RS pump infusion set failures. From the standard care group, one participant discontinued Dexcom G6 sensor use after delivery, and another switched (post-randomisation) to self-funded HCL therapy (CamAPS FX) starting during early pregnancy and continuing throughout the six-month postpartum period (Figure 1). One participant was missing baseline CGM data. In the HCL group, three participants were missing data in the 0-<3 months period and two were missing data in the 3-6 months period as assessed by CGM. In the standard care group, five participants were missing data in the 0-<3 months period and two were missing data in the 3-6 months period as assessed by CGM.

**Figure 2:**
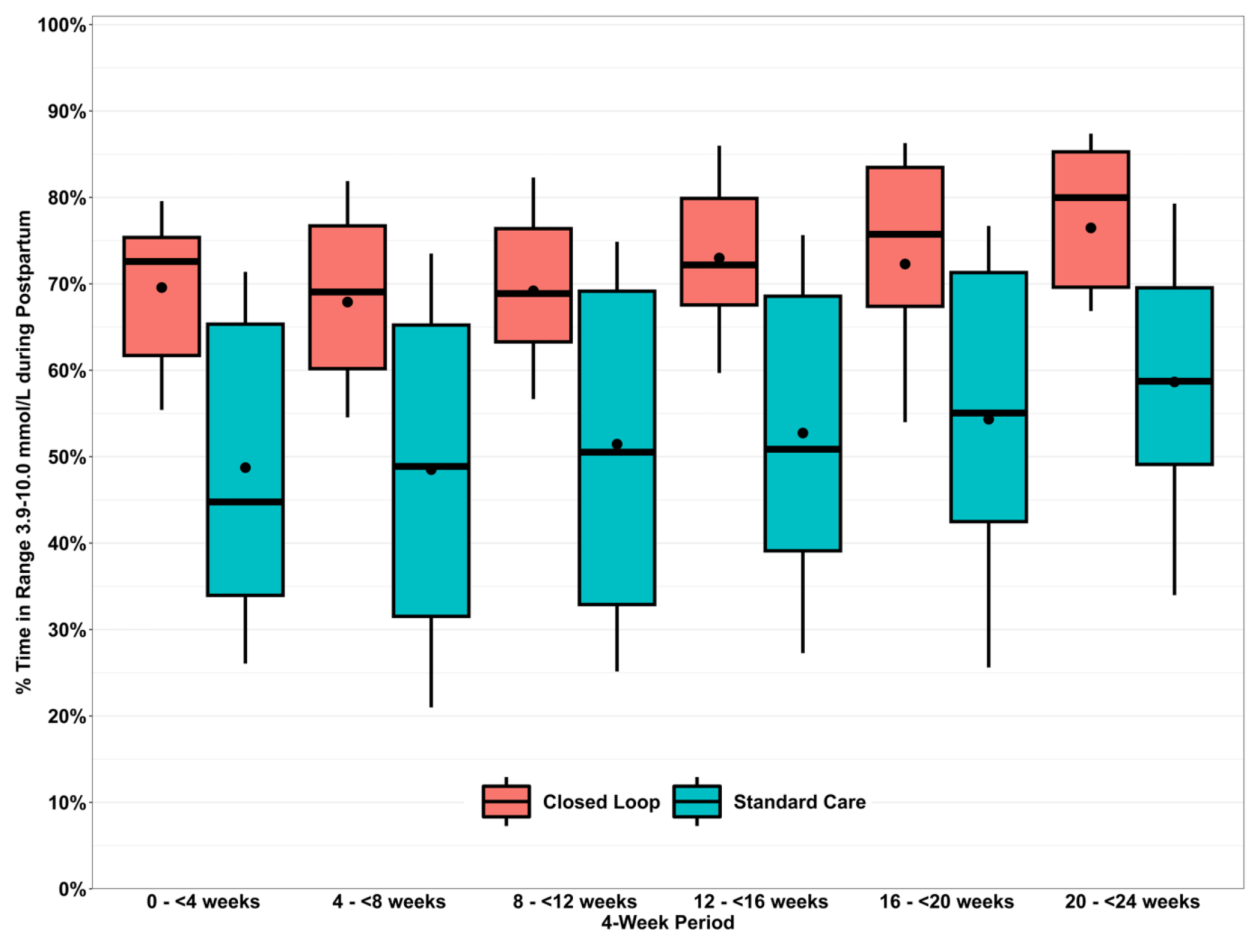
Time in target range 3.9-10.0mmol/L (70-180mg/dL) during the six months postpartum* *This figure shows TIR by 4-week period, and the TIR for the first 4-week period from day of delivery were 70% for HCL and 50% for standard care Dots are means, and the boxes are medians and quartiles. The whiskers are the 10^th^ and 90^th^ percentiles.

HCL users maintained mean percentage time in range (%TIR, 3.9-10.0mmol/L) from 73 ± 14% in early pregnancy (baseline run-in prior to randomisation) to 72 ± 12% throughout the six-month postpartum period (Table 2). For standard care participants, %TIR decreased from 70 ± 13% in early pregnancy to 54 ± 17% during the six-months postpartum. The mean adjusted treatment difference between the HCL intervention and standard care control group was 15% (95% CI 7 to 22%). Differences in glycaemia were apparent from the first four weeks postpartum and in each subsequent four-week period following delivery, with consistently higher TIR for the HCL group (Figure 2). Post hoc analysis of maternal glycaemia over the first 2 weeks postpartum starting from the day of delivery (requested during peer review) demonstrated the immediate impact of HCL vs standard insulin therapy with CGM use; TIR 71% (HCL) vs 51% (standard care).

The between-group treatment difference appeared similar when examined separately during the first three months after delivery and 4-6 months postpartum (16% [95% CI, 7 to 24%]; 15% [95% CI, 7 to 23%]) suggesting consistency of the treatment effect across both follow-up periods.

Associated glycaemic benefits of HCL use included lower mean glucose and less time spent above both the level 1 (10mmol/L, 180mg/dL) and level 2 (13.9mmol/L, 250mg/dL) hyperglycaemic thresholds (Table 2). The HCL group spent 14% [95% CI, -23% to -6%] less time >180mg/dL (>10.0mmol/L) during the first three months after delivery with sustained reductions over 4-6 months postpartum. Likewise, the HCL group had lower mean glucose levels during both follow-up periods: -1.5mmol/L (-2.6 to -0.3) -1.3mmol/L (-2.2 to -0.3) respectively. Hypoglycaemia rates were low, comparable between groups, and stable over the six-month follow-up period. There were temporal changes in glycaemic variability metrics consistent with higher glycaemic variation and less improvement in the standard care group at 3-6 months postpartum.

Overnight outcomes were similar to 24-hour results (Table 3). The overnight treatment difference was similar during the first three months and 4-6 months postpartum and achieved by marked reductions in mean glucose and nocturnal hyperglycaemia, without differences in hypoglycaemia. Insulin doses were consistent for each group for both months 0-<3 and 3-6 (Supplementary Table 2). Glycaemic outcomes were similar within HCL group regardless of insulin modality at baseline (Supplementary Table 3).

Adverse events were similar between the two groups. There was one severe hypoglycaemia event in standard care and none in the HCL group. There were no diabetic ketoacidosis episodes in either group during the six-month postpartum period. The rate of device-related adverse events in the HCL group was 7 per 100 person-years (Table 4).

Exclusive breastfeeding rates were lower in the HCL group at hospital discharge (39% vs 52%) and at 8-12 weeks postpartum (25% vs 43%), however breastfeeding rates were similar (36% vs. 42%) at six-months postpartum (Supplementary Table 4). Glycaemic outcomes were similar for formula and breast feeding participants in the HCL group, but for the standard insulin therapy group, those who fed their babies exclusively with breastmilk had better glycaemia than those feeding exclusively by formula (Supplementary Table 5).

In the lived experience feedback, standard care participants emphasised the challenges during the postpartum period and how this affected their diabetes management (Supplementary Table 6); “My glucose control during pregnancy was probably the best it had ever been, then since giving birth it’s been all over the show with the new (and huge) lifestyle changes, irregular eating patterns and breastfeeding” (SC11). HCL participants focussed more on the benefits of HCL (Supplementary Table 7); “Breastfeeding and sleepless nights were much easier to manage while on closed loop system. I had no concerns about my BG and was able to focus on my recovery and caring for a newborn” (HCL18). Having that mental headspace and freedom to not be thinking about my blood sugars all the time has allowed me to focus on my child and the value of that can’t be underestimated”(HCL13).

## Discussion

Women continuing HCL from pregnancy into the postpartum period spent 15% more time within the glucose target range (TIR 3.9-10.0mmol/L, 70-180mg/dL), an additional 3.6 hours a day, compared to those assigned to CGM alongside standard care insulin delivery. Glycaemic improvements were achieved by a marked reduction in maternal hyperglycaemia, especially evident overnight, and without additional hypoglycaemia.

The baseline glycaemic metrics obtained during early pregnancy at approximately 10 weeks’ gestation, were similar at 70% TIR 3.9-10.0mmol/L in both groups. Women assigned to HCL returned to target glycaemia (70% TIR) in the immediate postpartum period, whereas women assigned to standard care alongside real-time CGM, demonstrated a marked deterioration. The HCL treatment benefit was apparent from the first two weeks postpartum and consistently maintained over the six-month follow-up period. The first few weeks after birth, when women experience the most profound physiological and lifestyle transitions, often coincides with limited clinical input and oversight compared to the intensive support women receive during pregnancy.

Our results differ from the smaller Canadian CLIMB and American PICLS studies which described participants with lower baseline HbA1c: 52mmol/mol (6.9%) and 51mmol/mol (6.8%) and directly compared HCL with standard care over shorter time-frames (10 weeks and 4-6 weeks in the CLIMB and PICLS respectively). These two studies found continued optimal glycaemia both in HCL and in control arm participants using sensor-augmented pump therapy: CLIMB TIR 79% vs. 78% TIR and PICLS TIR 75% vs 77% TIR, without demonstrable clinical efficacy of HCL system use ^15,16^. Rates of hypoglycaemia were similar (~2%) among HCL participants between our AiDAPT trial participants and the CLIMB study but higher in the PICLS study (4.5%), most likely reflecting differences in baseline glycaemia and/or CGM sensors. It is notable that the CLIMB study participants commenced HCL use one week postpartum because of “concerns that the basal insulin modulation with the MiniMed 670/770G closed-loop system could be too strong for the first postpartum week”, reflecting HCL algorithm differences^15^. The MiniMed 670G/770G algorithm used by CLIMB participants is “strongly influenced by total daily dose of insulin used in the previous six days”, whereas previous evaluations of the intrapartum and first six weeks postpartum data supported continued use of CamAPS FX during labour, delivery and after immediately following birth^13,15^. The PICLS study, although examining the continuation of MiniMed 670G use from pregnancy, also stopped HCL automode during maternal hospital admissions for labour and delivery and resumed use three to seven days postpartum^16^. Potential limitations of these studies are their smaller sample sizes, delayed start and shorter duration of postnatal follow-up, limited statistical power to detect between group differences, and intensive postpartum follow-up visits schedule with monthly specialist endocrinology clinic visits and more study contacts including up to weekly remote glucose management in both. That level of intensive postpartum support is not representative of postpartum care in the UK^22^.

It is notable that whilst breastfeeding rates were initially low in the HCL group, they were similar between HCL and standard care groups by 6 months postpartum. Furthermore, these rates are comparable to national breastfeeding rates in the general maternity population, where prevalence of any breastfeeding is 55% at six weeks and 34% at six months postpartum^23^. Several factors beyond glycaemic control influence women’s infant feeding decision including maternal age, parity, BMI, socio-economic and educational status in addition to gestational age at birth and mode of delivery^24,25^. Our study was not designed to evaluate the complex interactions between maternal glycaemia, HCL therapy and infant feeding choices.

Strengths of this trial include its randomised design, larger sample size, generalisability of participants across a range of glycaemic categories, and inclusion of pump-naïve participants which is important for widening access to diabetes technology. Baseline characteristics of postpartum participants mirrored the overall characteristics of the main AiDAPT study which is highly representative of national population-based data for T1D pregnancy^10^. A further strength is the continuation of the same insulin delivery modality from pregnancy into the postpartum period, thereby eliminating any transition period between modalities which could affect maternal glycaemia. In this pragmatic postpartum extension study, there were no additional visits over and above usual clinical care. Limitations are that these postpartum results are specific to the CamAPS FX and cannot be extrapolated to other commercially available HCL systems. We designed this pragmatic postpartum extension study specifically not to add burden to healthcare teams in the immediate aftermath of the COVID-19 pandemic or to participants navigating life with a newborn baby. Therefore, we did not collect data including maternal weight or frequency of clinical postpartum contacts that were unavailable by maternal telephone contact. There is the possibility of measurement bias in the treatment estimates due to missing data, however, the number of participants missing was very low, with only four out of fifty-seven participants missing data for all periods. Additionally, unmeasured random confounding is a possible limitation, however, participants were randomized to their treatment groups and baseline characteristics appeared similar. Our study was not powered to examine specific HCL settings (insulin : carbohydrate ratios and personal glucose target), and the sample size is inadequate for examining complex interactions between maternal glycaemia, HCL therapy and infant feeding, or health economic analyses which all warrant future study. An evaluation of HCL therapy use during the inpatient admission for labour and delivery involving 119 participants will be reported separately.

The AiDAPT trial established the efficacy of HCL therapy during T1D pregnancy with glycaemic benefits over and above CGM with standard insulin therapy^10^. Our current findings support continued use of HCL from pregnancy into the postpartum period. Clinical benefits are sustained throughout the first six months postpartum compared to a marked deterioration in glycaemic control with CGM and standard insulin delivery. Provision and funding of healthcare is siloed into different streams and departments. For many patients this contributes to a sense of abandonment as they transition from team to team (maternity to adult diabetes care or general practice) with little to no continuity of care. This postpartum continuation of CamAPS FX HCL use allows mothers to maintain target glycaemic control whilst navigating clinical care transitions and adjusting to life with a newborn.

## Supplementary Material

Supplementary appendix

## Figures and Tables

**Table 1:** Participant Baseline Characteristics*

	Closed-Loop (N = 28)	Standard Care (N = 29)	Overall (N = 57)
**Age (years)**	32 ± 4	30 ± 4	31 ± 4
**Race N (%)** ^a^			
White	25 (89%)	25 (86%)	50 (88%)
**Booking BMI** ^b^	28.6 ± 4.5	25.8 ± 3.9	27.2 ± 4.4
**Education N (%)**			
Secondary education	3 (11%)	5 (17%)	8 (14%)
Further education	8 (29%)	9 (31%)	17 (30%)
University undergraduate degree or equivalent	14 (50%)	11 (38%)	25 (44%)
University postgraduate degree or equivalent	3 (11%)	4 (14%)	7 (12%)
**No previous births N (%)**			
0	7 (25%)	16 (55%)	23 (40%)
1	13 (46%)	11 (38%)	24 (42%)
2	6 (21%)	1 (3%)	7 (12%)
≥3	2 (7%)	1 (3%)	3 (5%)
**Diabetes Duration (years)**	17 ± 8	16 ± 7	16 ± 7
**HbA1c during early pregnancy**			
%	7.6 ± 1.1	7.6 ± 0.9	7.6 ± 1.0
mmol/mol	59.5 ± 11.6	59.3 ± 9.5	59.4 ± 10.5
**Diabetes complications**	15 (54%)	17 (59%)	32 (56%)
**Early pregnancy Insulin Modality**			
Pump	15 (54%)	11 (38%)	26 (46%)
Multiple dose injections	12 (43%)	17 (59%)	29 (51%)
Automated insulin delivery^d^	1 (4%)	1 (3%)	2 (4%)
**Adverse events pre-pregnancy** [No. of participants (IQR), previous 12 months]			
Pre-pregnancy DKA	0 (0%)	3 (10%)	3 (5%)
Previous severe hypoglycemia^c^	1 (4%)	2 (7%)	3 (5%)
**%Time in range 63-140 mg/dL during pregnancy** ^e^	67% ± 8%	58% ± 11%	62% ± 11%
**Median % Time CGM Use**^e^ (IQR)	96% (82%, 98%)	97% (94%, 98%)	97% (91%, 98%)
**Adverse events during pregnancy**^e^ (No. of events)			
Severe hypoglycaemia during pregnancy	5	1	6
DKA during pregnancy	1	1	2
**Maternal Weight Gain (kg)**	11.5 ± 6.1	15.3 ± 6.0	13.4 ± 6.3
**Pregnancy Duration at Delivery**	36.6 ± 1.7	37.0 ± 1.1	36.8 ± 1.4
**Mode of Delivery** [No. of participants (%)]			
Operative Vaginal	2 (7%)	2 (7%)	4 (7%)
Primary caesarean	8 (29%)	18 (62%)	26 (46%)
Repeat caesarean	14 (50%)	7 (24%)	21 (37%)
Vaginal	4 (14%)	2 (7%)	6 (11%)

* Plus- minus values are means ± SD. IQR denotes interquartile range.

a Race was reported by the participant.

b Body-mass index is the weight in kilograms divided by the square of the height in meters.

c Hypoglycaemia was considered severe if the event required third-party assistance.

d Participants using alternative hybrid closed-loop systems were eligible.

e 16 weeks’ until delivery

**Table 2:** Postnatal Maternal Glycaemic Outcomes by Treatment Group and 3-month Postpartum Period *

End Points	RCT Baseline^b^		Postpartum^c^		Adjusted Treatment Difference^a^ (95% CI)	P-value for Treatment Effect^a^	P-value for Interaction^a^
	Closed-Loop	Standard Care	Closed-Loop	Standard Care			
**Hours of CGM Data**			3,893 ± 622	3,636 ± 989			
**Number of participants** ^d^							
0-<3 months	N=25	N=24	N=25	N=24			
3-6 months	N=26	N=27	N=26	N=27			
**% Time 3.9-10.0 mmol/L**	73% ± 14%	70% ± 13%	72% ± 12%	54% ± 17%	15% (7%, 22%)	0.0037	0.83
0-<3 months	74% ± 13%	72% ± 13%	70% ± 9%	50% ± 19%	16% (7%, 24%)		
3-6 months	73% ± 14%	70% ± 13%	75% ± 12%	57% ± 16%	15% (7%, 23%)		
**% Time 3.9-7.8 mmol/L**	50% ± 15%	47% ± 12%	51% ± 11%	33% ± 13%	-	-	-
0-<3 months	51% ± 15%	48% ± 12%	48% ± 10%	30% ± 15%	-		
3-6 months	50% ± 15%	47% ± 12%	54% ± 12%	35% ± 12%	-		
**Mean Glucose (mg/dL)**	142 ± 24	142 ± 18	153 ± 26	180 ± 37	-23 (-41, -5)	0.036	0.78
0-<3 months	141 ± 23	140 ± 18	154 ± 18	189 ± 43	-26 (-46, -6)		
3-6 months	142 ± 24	142 ± 18	148 ± 27	174 ± 33	-23 (-40, -6)		
**Mean Glucose (mmol/L)**	7.9 ± 1.3	7.9 ± 1.0	8.5 ± 1.5	10.0 ± 2.0	-1.3 (-2.3, -0.3)	0.036	0.78
0-<3 months	7.8 ± 1.3	7.8 ± 1.0	8.6 ± 1.0	10.5 ± 2.4	-1.5 (-2.6, -0.3)		
3-6 months	7.9 ± 1.3	7.9 ± 1.0	8.2 ± 1.5	9.7 ± 1.9	-1.3 (-2.2, -0.3)		
**% Time >10.0 mmol/L**	22% ± 15%	22% ± 12%	26% ± 12%	42% ± 18%	-14% (-23%, -6%)	0.0055	0.78
0-<3 months	21% ± 15%	21% ± 12%	28% ± 10%	47% ± 21%	-15% (-25%, -6%)		
3-6 months	22% ± 15%	22% ± 12%	22% ± 13%	39% ± 17%	-15% (-23%, -6%)		
**Median % Time** **>13.9 mmol/L (IQR)**	3% (1%, 9%)	3% (1%, 7%)	7% (3%, 11%)	15% (8%, 30%)	-9% (-16%, -2%)	0.029	0.18
0-<3 months	2% (1%, 7%)	2% (1%, 6%)	8% (4%, 12%)	19% (9%, 37%)	-12% (-20%, -3%)		
3-6 months	3% (1%, 9%)	3% (1%, 7%)	4% (2%, 10%)	13% (6%, 22%)	-9% (-16%, -2%)		
**Median % Time** **<3.9 mmol/L (IQR)**	5.0% (3.1%, 6.8%)	4.6% (2.3%, 11.8%)	2.4% (1.5%, 4.0%)	2.6% (1.4%, 5.2%)	-0.7% (-2.0%, 0.5%)	0.49	0.78
0-<3 months	4.9% (3.1%, 6.8%)	4.0% (2.3%, 12.9%)	2.3% (1.6%, 3.7%)	2.2% (1.1%, 6.0%)	-0.6% (-2.0%, 0.7%)		
3-6 months	5.0% (3.1%, 6.8%)	4.6% (2.3%, 11.8%)	2.5% (1.4%, 4.3%)	2.9% (2.0%, 5.0%)	-0.8% (-2.2%, 0.6%)		
**Median % Time** **<3.0 mmol/L (IQR)**	1.2% (0.2%, 2.2%)	0.7% (0.3%, 3.1%)	0.4% (0.3%, 0.6%)	0.6% (0.2%, 1.3%)	-0.2% (-0.6%, 0.1%)	0.33	0.78
0-<3 months	1.0% (0.2%, 2.2%)	0.6% (0.3%, 3.1%)	0.4% (0.2%, 0.7%)	0.5% (0.2%, 1.5%)	-0.2% (-0.6%, 0.1%)		
3-6 months	1.2% (0.2%, 2.2%)	0.7% (0.3%, 3.1%)	0.4% (0.3%, 0.7%)	0.6% (0.2%, 1.2%)	-0.3% (-0.8%, 0.1%)		
**Glucose CV (%)**	36% ± 6%	37% ± 7%	39% ± 4%	40% ± 5%	0% (-2%, 3%)	0.012	0.019
0-<3 months	35% ± 5%	36% ± 7%	39% ± 4%	39% ± 6%	2% (-1%, 5%)		
3-6 months	36% ± 6%	37% ± 7%	37% ± 5%	40% ± 6%	-1% (-4%, 1%)		
**Glucose SD (mg/dL)**	51 ± 12	53 ± 13	60 ± 16	72 ± 17	-8 (-16, 1)	0.026	0.054
0-<3 months	50 ± 11	51 ± 12	61 ± 12	73 ± 18	-6 (-14, 2)		
3-6 months	51 ± 12	53 ± 13	56 ± 17	70 ± 17	-10 (-19, -1)		
**Glucose SD (mmol/L)**	2.8 ± 0.7	2.9 ± 0.7	3.3 ± 0.9	4.0 ± 1.0	-0.4 (-0.9, 0.0)	0.026	0.054
0-<3 months	2.8 ± 0.6	2.8 ± 0.7	3.4 ± 0.7	4.0 ± 1.0	-0.3 (-0.8, 0.1)		
3-6 months	2.8 ± 0.7	2.9 ± 0.7	3.1 ± 0.9	3.9 ± 1.0	-0.6 (-1.1, -0.1)		

* Plus-minus values are means ± SD. IQR denotes interquartile range.

a Based on a repeated measures linear regression model adjusting for baseline trial outcome, insulin delivery modality, and site as a random effect. Difference is Closed-Loop – Standard Care. P-values and confidence intervals adjusted using the adaptive Benjamini-Hochberg procedure.

b Baseline values were calculated with the use of data assessed by continuous glucose monitoring during the pre-randomisation run-in phase during early pregnancy. One participant was missing baseline data assessed by continuous glucose monitoring.

c The postpartum phase is from delivery until 24 weeks postpartum. Outcomes were assessed with the use of sensor data assessed by continuous glucose monitoring.

d In the HCL group, three participants had missing data in the 0-<3 months period and two had missing data in the 3-6 months period as assessed by continuous glucose monitoring. In the standard care group, five participants had missing data in the 0-<3 months period and two had missing data in the 3-6 months period as assessed by continuous glucose monitoring.

**Table 3:** Overnight Maternal Glycaemic Outcomes by Treatment Group and 3-month Postpartum Period *

End Points	RCT Baseline ^b^		Postpartum ^c^		Adjusted Treatment Difference ^a^ (95% CI)	P-value for Treatment Effect ^a^	P-value for Interaction ^a^
	Closed-Loop	Standard Care	Closed-Loop	Standard Care			
**Hours of CGM Data**			1,305 ± 207	1,208 ± 337			
**Number of participants^d^**							
0-<3 months	N=25	N=24	N=25	N=24			
3-6 months	N=26	N=27	N=26	N=27			
**% Time 3.9-10.0 mmol/L**	75% ± 19%	70% ± 14%	76% ± 12%	54% ± 17%	19% (11%, 27%)	0.0003	0.90
0-<3 months	76% ± 18%	70% ± 14%	74% ± 11%	50% ± 19%	20% (11%, 29%)		
3-6 months	75% ± 19%	70% ± 14%	79% ± 12%	57% ± 16%	19% (11%, 27%)		
**% Time 3.9-7.8 mmol/L**	53% ± 22%	47% ± 15%	55% ± 12%	33% ± 14%	-	-	-
0-<3 months	54% ± 21%	47% ± 15%	53% ± 12%	31% ± 16%	-		
3-6 months	53% ± 22%	47% ± 15%	57% ± 13%	35% ± 13%	-		
**Mean Glucose (mg/dL)**	142 ± 30	141 ± 19	149 ± 22	179 ± 34	-27 (-43, -11)	0.0069	0.77
0-<3 months	140 ± 28	142 ± 20	150 ± 18	186 ± 40	-30 (-49, -11)		
3-6 months	142 ± 30	141 ± 19	145 ± 23	174 ± 31	-26 (-42, -10)		
**Mean Glucose (mmol/L)**	7.9 ± 1.6	7.8 ± 1.1	8.3 ± 1.2	10.0 ± 1.9	-1.5 (-2.4, -0.6)	0.0069	0.77
0-<3 months	7.8 ± 1.6	7.9 ± 1.1	8.3 ± 1.0	10.3 ± 2.2	-1.7 (-2.7, -0.6)		
3-6 months	7.9 ± 1.6	7.8 ± 1.1	8.0 ± 1.3	9.7 ± 1.7	-1.4 (-2.3, -0.6)		
**% Time >10.0 mmol/L**	21% ± 20%	22% ± 12%	22% ± 11%	42% ± 17%	-18% (-26%, -10%)	0.0004	0.90
0-<3 months	20% ± 19%	22% ± 13%	24% ± 11%	46% ± 20%	-19% (-28%, -10%)		
3-6 months	21% ± 20%	22% ± 12%	19% ± 12%	39% ± 17%	-18% (-26%, -10%)		
**Median % Time** **>13.9 mmol/L (IQR)**	2% (0%, 9%)	3% (0%, 9%)	5% (2%, 9%)	16% (7%, 27%)	-13% (-20%, -4%)	0.0069	0.29
0-<3 months	2% (0%, 8%)	3% (0%, 9%)	6% (3%, 11%)	19% (9%, 34%)	-13% (-20%, -5%)		
3-6 months	2% (0%, 9%)	3% (0%, 9%)	4% (2%, 6%)	14% (6%, 22%)	-9% (-15%, -3%)		
**Median % Time** **<3.9 mmol/L (IQR)**	3.4% (1.2%, 6.5%)	6.0% (1.3%, 10.1%)	1.8% (1.1%, 2.9%)	3.3% (1.5%, 5.6%)	-1.3% (-2.6%, -0.0%)	0.10	0.90
0-<3 months	3.2% (1.2%, 6.5%)	5.1% (1.1%, 11.5%)	1.6% (1.0%, 2.6%)	2.8% (1.2%, 5.4%)	-1.3% (-2.5%, -0.1%)		
3-6 months	3.4% (1.2%, 6.5%)	5.1% (1.3%, 10.1%)	2.0% (1.0%, 2.9%)	3.1% (1.2%, 4.6%)	-1.5% (-3.1%, 0.0%)		
**Median % Time** **<3.0 mmol/L (IQR)**	0.2% (0.0%, 0.9%)	1.0% (0.0%, 2.4%)	0.4% (0.1%, 0.6%)	0.8% (0.2%, 1.5%)	-0.4% (-0.8%, 0.1%)	0.16	0.90
0-<3 months	0.1% (0.0%, 0.9%)	1.0% (0.0%, 2.4%)	0.3% (0.1%, 0.5%)	0.8% (0.1%, 1.5%)	-0.4% (-0.9%, 0.0%)		
3-6 months	0.2% (0.0%, 0.9%)	1.0% (0.0%, 2.4%)	0.3% (0.1%, 0.5%)	0.6% (0.1%, 1.4%)	-0.5% (-1.1%, 0.1%)		
**Glucose CV (%)**	33% ± 9%	36% ± 9%	37% ± 5%	40% ± 6%	-1% (-4%, 2%)	0.14	0.29
0-<3 months	32% ± 9%	36% ± 9%	37% ± 5%	39% ± 7%	-0% (-3%, 3%)		
3-6 months	33% ± 9%	36% ± 9%	36% ± 6%	40% ± 7%	-3% (-6%, 1%)		
**Glucose SD (mg/dL)**	47 ± 18	52 ± 15	56 ± 16	71 ± 18	-11 (-21, -2)	0.037	0.48
0-<3 months	46 ± 17	50 ± 14	57 ± 12	72 ± 18	-10 (-20, -1)		
3-6 months	47 ± 18	51 ± 14	53 ± 17	69 ± 19	-13 (-23, -3)		
**Glucose SD (mmol/L)**	2.6 ± 1.0	2.9 ± 0.8	3.1 ± 0.9	4.0 ± 1.0	-0.6 (-1.1, -0.1)	0.037	0.48
0-<3 months	2.6 ± 0.9	2.9 ± 0.8	3.1 ± 0.7	4.0 ± 1.0	-0.6 (-1.1, -0.1)		
3-6 months	2.6 ± 1.0	2.9 ± 0.8	2.9 ± 1.0	3.9 ± 1.0	-0.7 (-1.3, -0.2)		

* Plus-minus values are means ± SD. IQR denotes interquartile range.

a Based on a repeated measures linear regression model adjusting for baseline trial outcome, insulin delivery modality, and site as a random effect. Difference is Closed-Loop – Standard Care. P-values and confidence intervals adjusted using the adaptive Benjamini-Hochberg procedure.

b Baseline values were calculated with the use of data assessed by continuous glucose monitoring during the pre-randomization run-in phase during early pregnancy. One participant was missing baseline data assessed by continuous glucose monitoring.

c The postpartum phase is from delivery until 24 weeks postpartum. Outcomes were assessed with the use of sensor data assessed by continuous glucose monitoring.

d In the HCL group, three participants had missing data in the 0-<3 months period and two had missing data in the 3-6 months period as assessed by continuous glucose monitoring. In the standard care group, five participants had missing data in the 0-<3 months period and two had missing data in the 3-6 months period as assessed by continuous glucose monitoring.

**Table 4:** Adverse Events

	Closed-Loop (N = 28)	Standard Care (N = 29)
**Severe Hypoglycaemia**		
Number of events	0	1
Participants with ≥1 event	0 (0%)	1 (3%)
Incidence per 100 person years	0.0	7.0
**Hyperglycaemia with ketosis**		
Number of events	0	0
Mild to moderate*	3	0
Severe†	0	1
Diabetic ketoacidosis‡	0	0
Participants with ≥1 event	2 (7%)	1 (3%)
Incidence of diabetic ketoacidosis per 100 person years	0.0	0.0
**Serious AE** §		
Number of events	2	6
Hypoglycaemia	0	2
Hyperglycaemia with ketosis	0	1
Other	2	3
Participants with ≥1 event	1 (4%)	6 (21%)
Incidence per 100 person years	14.5	42.0
**Device-related adverse events with the closed-loop system**		
Number of events¶	1	0
Participants with ≥1 event	1 (4%)	0 (0%)
Incidence per 100 person years	7.0	0.0
**Device-related adverse events with the continuous glucose monitor**		
Number of events	0	0
Participants with ≥1 event	0 (0%)	0 (0%)
Incidence per 100 person years	0.0	0.0

* Mild-to-moderate events include ketosis (ketones >0.5 mmol per litre) that were treated by the participant and resolved without hospital admission.

† Severe ketosis was defined as a level of plasma ketones above 1.0 mmol per litre that resulted in hospital admission and treatment with intravenous insulin. One participant had 20 events, none of which occurred while using closed-loop therapy.

‡ Diabetic ketoacidosis was defined as ketosis with acidosis that resulted in treatment with fixed-rate intravenous insulin infusion.

§ Serious adverse events were defined as adverse events that resulted in death, a serious deterioration in health, life-threatening illness or injury, permanent impairment, in-patient or prolonged hospitalization

¶ There was 1 device-related adverse events occurring in the closed-loop group. This was due to a pump infusion set failure (kinked cannula) resulting in hyperglycaemia without ketosis.

## Data Availability

The data that support the findings were used under license for the current study and therefore are not publicly available. Data are however available upon reasonable request and after completion of a Data User Agreement form.

## Research in context

### Evidence before this study

We searched PubMed for articles published before 12 June 2024 without restriction on language or start date. We included the search terms (“diabetes mellitus” OR “diabetes”) AND (“pregnancy” OR “postpartum” OR “postnatal”) AND (“closed loop” OR “automated insulin delivery”).

We identified five case reports or case series and an additional four studies of commercially available hybrid closed-loop (HCL) systems in pregnancy which examined their use in the postpartum period. In three of the four studies, HCL was continued from pregnancy, whilst in the last, HCL was commenced one week after birth. Whilst these studies demonstrated good glycaemic outcomes associated with HCL use, >70% time in range (TIR 3.9-10.0mmol/L) and low rates of hypoglycaemia, there was no improvement in glycaemic outcomes compared with sensor-augmented pump therapy in the two randomised trials examining the Medtronic MiniMed HCL systems.

Given the limited size and scope of existing studies of postpartum HCL use, current NICE guidance in the United Kingdom does not specifically address diabetes management in the postpartum period including postpartum continuation of HCL.

### Added value of this study

This study is the largest study of HCL use in the postpartum period. 57 out of 58 eligible participants with type 1 diabetes continued their assigned diabetes management: HCL or standard care (insulin pump / multiple daily injections with CGM) following randomisation in early pregnancy. We found that CamAPS FX HCL users spent more time in the target glycaemic range (3.9-10.0mmol/L) over the six-month postpartum period compared to those continuing standard care (insulin pump / multiple daily injections) with no increase in hypoglycaemia rates. Trial participants spanned a range of glycaemia categories, were representative of the UK T1D population and over half were pump-naïve.

### Implications of all the available evidence

Our study demonstrates sustained gycaemic benefits of HCL use from pregnancy into the postpartum period. Women continuing HCL returned to non-pregnancy target glycaemia during both the immediate and six-month postpartum periods, whilst those using standard care experienced a marked (~50%) glycaemic deterioration. These findings support the continued use of HCL into the postpartum period, when clinical care is fragmented, and diabetes self-management is challenged by the 24/7 demands of caring for a newborn.
