# Supplementary material for: Automated insulin delivery during the first six months postpartum: pre-specified AiDAPT extension study
Source: Lancet Diabetes Endocrinol. Author manuscript; Available in PMC 2025 Jun 21. (PMC7617783; doi:10.1016/S2213-8587(24)00340-1)
Supplement: Supplementary appendix [file EMS205678-supplement-Supplementary_appendix.pdf]

THE LANCET
Diabetes & Endocrinology

Supplementary appendix

This appendix formed part of the original submission and has been peer reviewed. We post it as supplied by the authors.

Supplement to: Lee TTM, Collett C, Bergford S, et al. Automated insulin delivery during the first 6 months postpartum (AiDAPT): a prespecified extension study. *Lancet Diabetes Endocrinol* 2025; published online Jan 27. https://doi.org/10.1016/S2213-8587(24)00340-1.

Supplementary Appendix

Table of Contents

AiDAPT Investigators 1

Additional Qualitative Methods Details 2

Supplementary Figure 1: Flow of Participants through Postpartum Study 3

Supplementary Table 1: Characteristics of Included and Ineligible Postpartum Study Participants 4

Supplementary Table 2: Overnight Maternal Glycaemic Outcomes by Treatment Group and 3-month Postpartum Period 6

Supplementary Table 3: Postnatal Glycaemic Outcomes by Treatment Group During First 2 Weeks Postpartum 8

Supplementary Table 4: Insulin Use and Personal Glucose Targets 8

Supplementary Table 5: Postnatal Glycaemic Outcomes by Baseline Insulin Modality 9

Supplementary Table 6: Infant Feeding 11

Supplementary Table 7: Postnatal Glycaemic Outcomes by Infant Feeding Method 12

Supplementary Table 8: Participant Feedback – Standard care participants 14

Supplementary Table 9: Participant Feedback – Intervention participants 16

AiDAPT Investigators (AiDAPT Collaborative Group Members)

Katharine F Hunt, Helen Rogers, **King's College Hospital**, *London, UK.*

Damian Morris, Duncan Fowler, Josephine Rosier, Zeenat Banu, Sarah Barker, Gerry Rayman **Ipswich Hospital NHS Trust**, *Ipswich, UK.*

Eleanor Gurnell, Caroline Byrne, Andrea Lake, Katy Davenport, Jeannie Grisoni, Shannon Savine **Cambridge University Hospitals NHS Foundation Trust**, *Cambridge*, *UK.*

Helen R Murphy, Tara TM Lee, Tara Wallace, Alastair McKelvey, Nina Willer, **Norfolk and Norwich University Hospital NHS Foundation Trust**, *Norwich, UK.*

Corinne Collett, Mei-See Man, Emma Flanagan, Matt Hammond, Lee Shepstone, **Norwich Clinical Trials Unit,** *Norwich, UK.*

Anna Brackenridge, Sara White, Anna Reid, Olanike Okolo, **Guys and St Thomas' NHS Foundation Trust**, *London, UK.*

Eleanor M Scott, Del Endersby **Leeds Teaching Hospitals NHS Foundation Trust**, *Leeds, UK.*

Anna Dover, Frances Dougherty, Susan Johnston, Rebecca M Reynolds, **Royal Infirmary of Edinburgh**, *Edinburgh, UK.*

Robert S Lindsay, David Carty, Sharon Mackin, Isobel Crawford, Ross Buchan, **Glasgow Royal Infirmary**, *Glasgow, UK.*

David R McCance, Louisa Jones, Joanne Quinn, **Belfast Health and Social Care Trust**, *Belfast, Northern Ireland.*

Sarah Cains, Goher Ayman **Patient and Public Involvement (PPI) Leads.**

Additional Qualitative Methods Details

Data collection

To assess diabetes and treatment-related lived experience as described through self-reported free text feedback, women were sent the following questions to answer.

At the 8-12 week postpartum virtual follow up visit:

"Thinking about your use of the CGM or closed-loop artificial pancreas, please could you tell us what impact you think it had on the following areas:

- Your feelings about your blood glucose levels, if you used the CGM or closed-loop system, in the six weeks after giving birth
- Thoughts around safety for your health and the health of your baby if you used the CGM or closed-loop, in the six weeks after giving birth"

At the 24 week (6 month) postpartum virtual follow up visit:

"Thinking about your use of the CGM or closed-loop artificial pancreas, please could you tell us what impact you think it had on the following areas:

- Your feelings about your blood glucose levels, if you used the CGM or closed-loop system, in the six months after giving birth

Thoughts around safety for your health and the health of your baby if you used the CGM or closed-loop, in the six months after giving birth"

Data analysis

Data analysis sought to identify descriptive and analytical themes with relevance to clinical practice. The written responses from control and intervention participants were separated and then read through and cross-compared to identify common themes. Coding was used to capture data relevant to each of these themes and subsequent coded reports were used to identify quotes. The qualitative software Qualcoder version 3.5 was used to facilitate data coding and retrieval.

Supplementary Figure 1: Flow of Participants through Postpartum Study

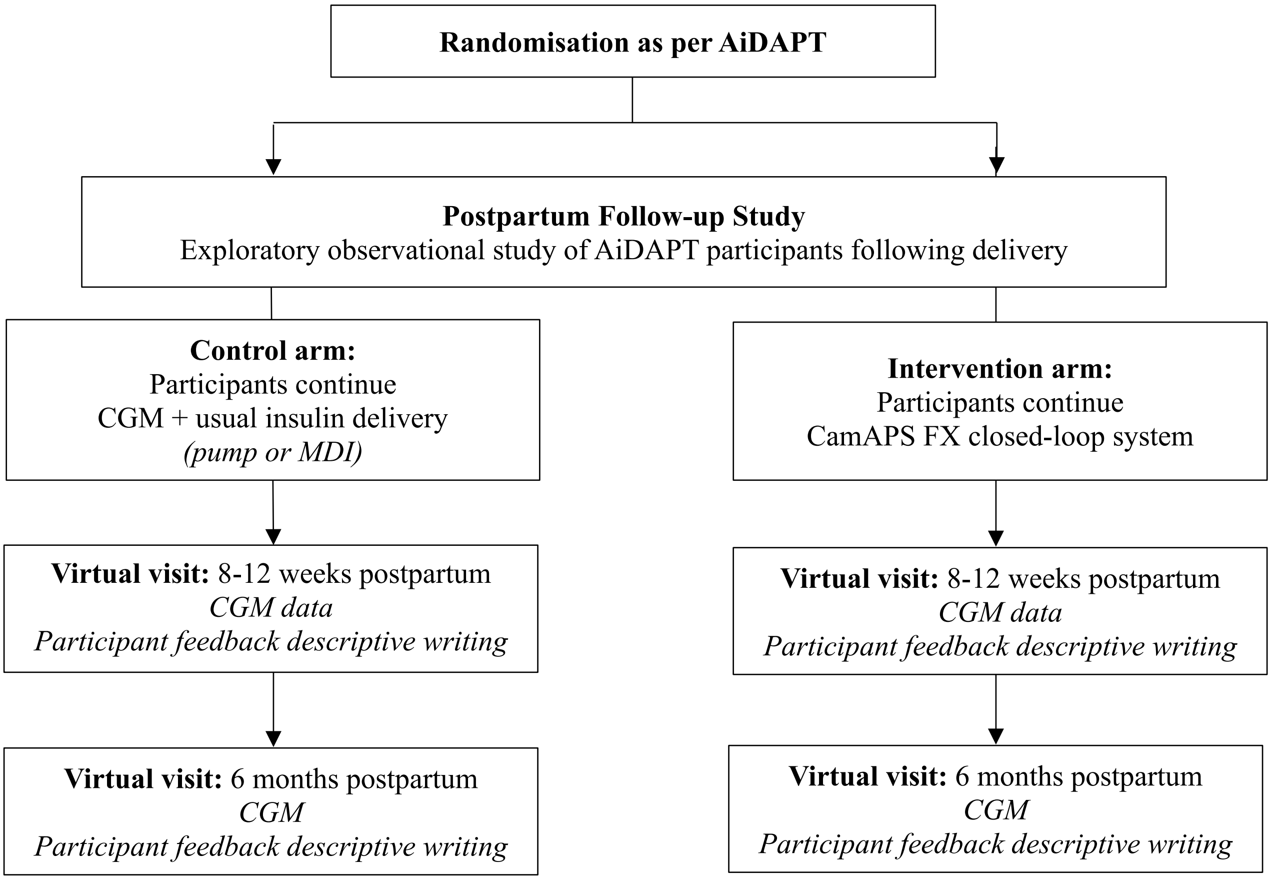

Supplementary Table 1. Characteristics of Included and Ineligible Postpartum Study Participants*

	Postpartum Participants		Ineligible Participants[a]	
	Hybrid closed-loop (N = 28)	Standard Care (N = 29)	Hybrid closed-loop (N = 33)	Standard Care (N = 34)
**Age (years)**	32 ± 4	30 ± 4	32 ± 6	30 ± 6
**Race N (%)[b]**				
White	25 (89%)	25 (86%)	33 (100%)	32 (94%)
**Booking BMI[c]**	28.6 ± 4.5	25.8 ± 3.9	27.3 ± 6.8	27.9 ± 5.3
**Education N (%)**				
Secondary education	3 (11%)	5 (17%)	4 (12%)	5 (15%)
Further education	8 (29%)	9 (31%)	10 (30%)	11 (32%)
University undergraduate degree or equivalent	14 (50%)	11 (38%)	11 (33%)	13 (38%)
University postgraduate degree or equivalent	3 (11%)	4 (14%)	8 (24%)	5 (15%)
**No previous births N (%)**				
0	7 (25%)	16 (55%)	14 (42%)	22 (65%)
1	13 (46%)	11 (38%)	10 (30%)	10 (29%)
2	6 (21%)	1 (3%)	8 (24%)	2 (6%)
≥3	2 (7%)	1 (3%)	3 (5%)	0 (0%)
**Diabetes Duration (years)**	17 ± 8	16 ± 7	19 ± 8	16 ± 8
**HbA1c during early pregnancy**				
%	7.6 ± 1.1	7.6 ± 0.9	7.6 ± 1.2	8.2 ± 1.5
mmol/mol	59.5 ± 11.6	59.3 ± 9.5	59.1 ± 12.7	65.8 ± 16.1
**Diabetes complications**	15 (54%)	17 (59%)	20 (61%)	18 (53%)
**Early pregnancy Insulin Modality**				
Pump	15 (54%)	11 (38%)	17 (52%)	14 (41%)
Multiple dose injections	12 (43%)	17 (59%)	15 (45%)	20 (59%)
Automated insulin delivery[d]	1 (4%)	1 (3%)	1 (3%)	0
**Adverse events pre-pregnancy**				
[No. of participants (%), previous 12 months]				
Pre-pregnancy DKA	0 (0%)	3 (10%)	1 (3%)	7 (21%)
Previous severe hypoglycaemia[e]	1 (4%)	2 (7%)	3 (9%)	3 (9%)
**%Time in range 3.5 – 7.8mmol/L (63-140 mg/dL) during pregnancy[f]**	67% ± 8%	58% ± 11%	69% ± 12%	54% ± 14%
**Median % Time CGM Use[f]** (IQR)	96% (82%, 98%)	97% (94%, 98%)	97% (96%, 98%)	95% (90%, 98%)
**Maternal Weight Gain (kg)**	11.5 ± 6.1	15.3 ± 6.0	10.7 ± 6.2	12.4 ± 6.0
**Pregnancy Duration at Delivery**	36.6 ± 1.7	37.0 ± 1.1	36.4 ± 1.7	37.2 ± 1.3

**Mode of Delivery**				
No delivery	0 (0%)	0 (0%)	2 (6%)	3 (9%)
Operative Vaginal	2 (7%)	2 (7%)	1 (3%)	3 (9%)
Primary caesarean	8 (29%)	18 (62%)	16 (48%)	16 (47%)
Repeat caesarean	14 (50%)	7 (24%)	11 (33%)	4 (12%)
Vaginal	4 (14%)	2 (7%)	3 (9%)	8 (24%)

* Plus- minus values are means ± SD. IQR denotes interquartile range.

a Reasons for not meeting trial eligibility criteria were:

- Participants more than 6 months postpartum (N=60)
- Completed the study and returned to NHS care prior to extension being implemented and so these participants were no longer using hybrid closed-loop (CamAPS FX) or continuous glucose monitoring (Dexcom G6). (N=6)

This also includes a participant who had a neonatal death and it was felt by both trial team and local site investigators inappropriate to approach to continue into postnatal extension.

b Race was reported by the participant.

c Body-mass index is the weight in kilograms divided by the square of the height in meters.

d Participants using alternative hybrid closed-loop systems were eligible.

e Hypoglycaemia was considered severe if the event required third-party assistance.

f 16 weeks' until delivery

Supplementary Table 2. Overnight Maternal Glycaemic Outcomes by Treatment Group and 3-month Postpartum Period *

End Points	RCT Baseline [b]		Postpartum [c]		Adjusted Treatment Difference [a] (95% CI)	P-value for Treatment Effect [a]	P-value for Interaction [a]
	Hybrid closed-loop	Standard Care	Hybrid closed-loop	Standard Care			
**Hours of CGM Data**			1,305 ± 207	1,208 ± 337			
**Number of participants[d]**							
0-<3 months	N=26	N=27	N=26	N=27			
3-6 months	N=25	N=24	N=25	N=24			
**% Time 3.9-10.0 mmol/L**	75% ± 19%	70% ± 14%	76% ± 12%	54% ± 17%	19% (11%, 27%)	0.0003	0.90
0-<3 months	75% ± 19%	70% ± 14%	79% ± 12%	57% ± 16%	19% (11%, 27%)		
3-6 months	76% ± 18%	70% ± 14%	74% ± 11%	50% ± 19%	20% (11%, 29%)		
**% Time 3.9-7.8 mmol/L**	53% ± 22%	47% ± 15%	55% ± 12%	33% ± 14%	-	-	-
0-<3 months	53% ± 22%	47% ± 15%	57% ± 13%	35% ± 13%	-		
3-6 months	54% ± 21%	47% ± 15%	53% ± 12%	31% ± 16%	-		
**Mean Glucose (mg/dL)**	142 ± 30	141 ± 19	149 ± 22	179 ± 34	-27 (-43, -11)	0.0069	0.77
0-<3 months	142 ± 30	141 ± 19	145 ± 23	174 ± 31	-26 (-42, -10)		
3-6 months	140 ± 28	142 ± 20	150 ± 18	186 ± 40	-30 (-49, -11)		
**Mean Glucose (mmol/L)**	7.9 ± 1.6	7.8 ± 1.1	8.3 ± 1.2	10.0 ± 1.9	-1.5 (-2.4, -0.6)	0.0069	0.77
0-<3 months	7.9 ± 1.6	7.8 ± 1.1	8.0 ± 1.3	9.7 ± 1.7	-1.4 (-2.3, -0.6)		
3-6 months	7.8 ± 1.6	7.9 ± 1.1	8.3 ± 1.0	10.3 ± 2.2	-1.7 (-2.7, -0.6)		
**% Time >10.0 mmol/L**	21% ± 20%	22% ± 12%	22% ± 11%	42% ± 17%	-18% (-26%, -10%)	0.0004	0.90
0-<3 months	21% ± 20%	22% ± 12%	19% ± 12%	39% ± 17%	-18% (-26%, -10%)		
3-6 months	20% ± 19%	22% ± 13%	24% ± 11%	46% ± 20%	-19% (-28%, -10%)		

**Median % Time >13.9 mmol/L (IQR)**	2% (0%, 9%)	3% (0%, 9%)	5% (2%, 9%)	16% (7%, 27%)	-13% (-20%, -4%)	0.0069	0.29
0-<3 months	2% (0%, 9%)	3% (0%, 9%)	4% (2%, 6%)	14% (6%, 22%)	-9% (-15%, -3%)		
3-6 months	2% (0%, 8%)	3% (0%, 9%)	6% (3%, 11%)	19% (9%, 34%)	-13% (-20%, -5%)		
**Median % Time <3.9 mmol/L (IQR)**	3.4% (1.2%, 6.5%)	6.0% (1.3%, 10.1%)	1.8% (1.1%, 2.9%)	3.3% (1.5%, 5.6%)	-1.3% (-2.6%, -0.0%)	0.10	0.90
0-<3 months	3.4% (1.2%, 6.5%)	5.1% (1.3%, 10.1%)	2.0% (1.0%, 2.9%)	3.1% (1.2%, 4.6%)	-1.5% (-3.1%, 0.0%)		
3-6 months	3.2% (1.2%, 6.5%)	5.1% (1.1%, 11.5%)	1.6% (1.0%, 2.6%)	2.8% (1.2%, 5.4%)	-1.3% (-2.5%, -0.1%)		
**Median % Time <3.0 mmol/L (IQR)**	0.2% (0.0%, 0.9%)	1.0% (0.0%, 2.4%)	0.4% (0.1%, 0.6%)	0.8% (0.2%, 1.5%)	-0.4% (-0.8%, 0.1%)	0.16	0.90
0-<3 months	0.2% (0.0%, 0.9%)	1.0% (0.0%, 2.4%)	0.3% (0.1%, 0.5%)	0.6% (0.1%, 1.4%)	-0.5% (-1.1%, 0.1%)		
3-6 months	0.1% (0.0%, 0.9%)	1.0% (0.0%, 2.4%)	0.3% (0.1%, 0.5%)	0.8% (0.1%, 1.5%)	-0.4% (-0.9%, 0.0%)		
**Glucose CV (%)**	33% ± 9%	36% ± 9%	37% ± 5%	40% ± 6%	-1% (-4%, 2%)	0.14	0.29
0-<3 months	33% ± 9%	36% ± 9%	36% ± 6%	40% ± 7%	-3% (-6%, 1%)		
3-6 months	32% ± 9%	36% ± 9%	37% ± 5%	39% ± 7%	-0% (-3%, 3%)		
**Glucose SD (mg/dL)**	47 ± 18	52 ± 15	56 ± 16	71 ± 18	-11 (-21, -2)	0.037	0.48
0-<3 months	47 ± 18	51 ± 14	53 ± 17	69 ± 19	-13 (-23, -3)		
3-6 months	46 ± 17	50 ± 14	57 ± 12	72 ± 18	-10 (-20, -1)		
**Glucose SD (mmol/L)**	2.6 ± 1.0	2.9 ± 0.8	3.1 ± 0.9	4.0 ± 1.0	-0.6 (-1.1, -0.1)	0.037	0.48
0-<3 months	2.6 ± 1.0	2.9 ± 0.8	2.9 ± 1.0	3.9 ± 1.0	-0.7 (-1.3, -0.2)		
3-6 months	2.6 ± 0.9	2.9 ± 0.8	3.1 ± 0.7	4.0 ± 1.0	-0.6 (-1.1, -0.1)		

* Plus-minus values are means ± SD. IQR denotes interquartile range.
a Based on a repeated measures linear regression model adjusting for baseline trial outcome, insulin delivery modality, and site as a random effect. Difference is Closed-Loop – Standard Care. P-values and confidence intervals adjusted using the adaptive Benjamini-Hochberg procedure.
b Baseline values were calculated with the use of data assessed by continuous glucose monitoring during the pre-randomization run-in phase during early pregnancy. One participant was missing baseline data assessed by continuous glucose monitoring.
c The postpartum phase is from delivery until 24 weeks postpartum. Outcomes were assessed with the use of sensor data assessed by continuous glucose monitoring.
d In the HCL group, three participants had missing data in the 0-<3 months period and two had missing data in the 3-6 months period as assessed by continuous glucose monitoring. In the standard care group, five participants had missing data in the 0-<3 months period and two had missing data in the 3-6 months period as assessed by continuous glucose monitoring.

Supplementary Table 3: Postnatal Glycaemic Outcomes by Treatment Group During First 2 Weeks of Postpartum*

End Points	Postpartum	
	Hybrid closed-loop	Standard Care
**Number of participants**	N=26	N=27
**% Time 3.9-10.0 mmol/L**	80% ± 9%	67% ± 14%

* Plus-minus values are means ± SD.

Supplementary Table 4: Insulin Use and Personal Glucose Targets

	0-<3 months		3-6 months	
	Hybrid closed-loop	Standard Care	Hybrid closed-loop	Standard Care
**Total Daily Insulin***	N = 27	N = 27	N = 27	N = 25
**Units / day** Median (IQR)	43 (38, 61)	36 (28, 45)	44 (29, 54)	40 (34, 47)
**Daily Basal Insulin**	N =27	N = 27	N =27	N =25
**Units / day** Median (IQR)	28 (19, 43)	19 (16, 23)	26 (17, 38)	23 (18, 26)
**Daily Bolus Insulin**	N =27	N =23	N =27	N =23
**Units / day** Median (IQR)	17 (10, 22)	20 (13, 27)	14 (11, 22)	18 (13, 24)
**Personal Glucose Target**	N = 28	-	N =28	-
**mmol/L** Mean ± SD	5.92 ± 0.34	-	5.89 ± 0.41	-

* In the HCL group, one participant was missing total daily, daily basal and daily bolus insulin data in the 0-<3 months period and the 3-6 months period.
In the standard care group, two participants were missing total daily and daily basal insulin data in the 0-<3 months period and four participants in the 3-6 months period. Six participants were missing daily bolus insulin data in the 0-<3 months period and the 3-6 months period.

Supplementary Table 5: Postnatal Glycaemic Outcomes by Baseline Insulin Modality *

End Points	Hybrid closed-loop			Standard Care		
	Pump	MDI	AID	Pump	MDI	AID
**Number of participants**						
0-<3 months	N=15	N=10	N=1	N=11	N=15	N=1
3-6 months	N=14	N=10	N=1	N=10	N=14	N=0
**% Time 3.9-10.0 mmol/L**						
0-<3 months	74% ± 14%	76% ± 10%	68%	57% ± 16%	56% ± 16%	73%
3-6 months	71% ± 8%	68% ± 11%	57%	52% ± 17%	48% ± 21%	-
**Mean Glucose (mg/dL)**						
0-<3 months	150 ± 32	145 ± 19	146	168 ± 33	180 ± 35	149
3-6 months	151 ± 14	157 ± 22	175	177 ± 37	197 ± 46	-
**Mean Glucose (mmol/L)**						
0-<3 months	8.3 ± 1.8	8.1 ± 1.1	8.1	9.4 ± 1.8	10.0 ± 1.9	8.3
3-6 months	8.4 ± 0.8	8.7 ± 1.2	9.7	9.9 ± 2.0	10.9 ± 2.6	-
**% Time >10.0 mmol/L**						
0-<3 months	23% ± 14%	21% ± 11%	24%	38% ± 18%	41% ± 16%	24%
3-6 months	26% ± 8%	29% ± 12%	39%	42% ± 19%	50% ± 22%	-
**Median % Time >13.9 mmol/L (IQR)**						
0-<3 months	3% (2%, 10%)	4% (2%, 7%)	10%	13% (6%, 19%)	16% (7%, 25%)	4%
3-6 months	5% (3%, 11%)	10% (4%, 13%)	20%	18% (6%, 24%)	21% (10%, 42%)	-
**Median % Time <3.9 mmol/L (IQR)**						
0-<3 months	2.1% (1.4%, 4.3%)	2.6% (1.0%, 3.8%)	7.6%	5.0% (2.6%, 5.6%)	2.7% (1.8%, 3.1%)	2.5%
3-6 months	2.1% (1.6%, 3.7%)	2.4% (0.8%, 3.5%)	4.5%	5.4% (1.7%, 9.3%)	1.6% (1.0%, 3.4%)	-

**Median % Time <3.0 mmol/L (IQR)**						
0-<3 months	0.4% (0.3%, 0.5%)	0.4% (0.2%, 0.9%)	2.2%	1.1% (0.1%, 1.7%)	0.5% (0.2%, 1.0%)	0.4%
3-6 months	0.4% (0.2%, 0.7%)	0.4% (0.2%, 0.6%)	1.1%	1.5% (0.2%, 2.3%)	0.5% (0.1%, 0.6%)	-
**Glucose CV (%)**						
0-<3 months	37% ± 4%	37% ± 5%	49%	40% ± 5%	40% ± 6%	36%
3-6 months	38% ± 4%	40% ± 3%	50%	41% ± 7%	37% ± 6%	-
**Glucose SD (mg/dL)**						
0-<3 months	56 ± 19	54 ± 12	72	67 ± 12	73 ± 20	53
3-6 months	57 ± 10	63 ± 11	88	72 ± 15	73 ± 20	-
**Glucose SD (mmol/L)**						
0-<3 months	3.1 ± 1.1	3.0 ± 0.7	4.0	3.7 ± 0.7	4.1 ± 1.1	3.0
3-6 months	3.2 ± 0.6	3.5 ± 0.6	4.9	4.0 ± 0.8	4.1 ± 1.1	-

* Plus-minus values are means ± SD. IQR denotes interquartile range.
MDI = multiple daily injections. AID = automated insulin delivery

Supplementary Table 6: Infant Feeding

	Hybrid closed-loop	Standard Care
**Feeding at time of hospital discharge after delivery**		
Exclusive breastfeeding[a] / breast milk	11 (39%)	15 (52%)
Breast milk and formula	7 (25%)	9 (31%)
Exclusive formula feeding	10 (36%)	5 (17%)
**Feeding at 8-12 weeks postnatal**		
Exclusive breastfeeding[a] / breast milk	7 (25%)	12 (43%)
Breast milk and formula	7 (25%)	5 (18%)
Exclusive formula feeding	14 (50%)	11 (39%)
**Feeding at 24 weeks postnatal**		
Exclusive breastfeeding[a] / breast milk	10 (36%)	11 (42%)
Breast milk and formula	2 (7%)	2 (8%)
Exclusive formula feeding	16 (57%)	13 (50%)

a Exclusive breastfeeding as defined by the World Health Organization as “receiving only breast milk. No other liquids or solids are given – not even water – with the exception of oral rehydration solution, or drops/syrups of vitamins, minerals or medicines”.

Supplementary Table 7: Postnatal Glycaemic Outcomes by Infant Feeding Method *

End Points	Exclusive Breastfeeding		Mixed Feeding: Breast Milk and Formula		Exclusive Formula Feeding	
	Hybrid closed-loop	Standard Care	Hybrid closed-loop	Standard Care	Hybrid closed-loop	Standard Care
**Number of participants**						
0-<3 months	N=7	N=11	N=5	N=5	N=14	N=11
3-6 months	N=8	N=10	N=1	N=2	N=16	N=11
**% Time 3.9-10.0 mmol/L**						
0-<3 months	80% ± 9%	66% ± 14%	75% ± 14%	45% ± 12%	72% ± 13%	54% ± 15%
3-6 months	69% ± 11%	60% ± 16%	81%	38% ± 10%	69% ± 8%	44% ± 20%
**Mean Glucose (mg/dL)**						
0-<3 months	134 ± 14	155 ± 23	148 ± 26	198 ± 24	155 ± 31	182 ± 38
3-6 months	155 ± 20	165 ± 30	123	207 ± 19	156 ± 16	202 ± 47
**Mean Glucose (mmol/L)**						
0-<3 months	7.4 ± 0.8	8.6 ± 1.3	8.2 ± 1.4	11.0 ± 1.4	8.6 ± 1.7	10.1 ± 2.1
3-6 months	8.6 ± 1.1	9.1 ± 1.7	6.8	11.5 ± 1.1	8.6 ± 0.9	11.2 ± 2.6
**% Time >10.0 mmol/L**						
0-<3 months	16% ± 9%	29% ± 14%	23% ± 14%	54% ± 13%	25% ± 13%	42% ± 15%
3-6 months	28% ± 11%	35% ± 17%	11%	61% ± 10%	29% ± 9%	53% ± 21%
**Median % Time >13.9 mmol/L (IQR)**						
0-<3 months	2% (1%, 9%)	7% (4%, 13%)	5% (2%, 10%)	20% (20%, 25%)	5% (3%, 11%)	11% (8%, 31%)
3-6 months	9% (3%, 13%)	12% (5%, 20%)	2%	27% (20%, 35%)	9% (4%, 11%)	25% (10%, 46%)
**Median % Time <3.9 mmol/L (IQR)**						
0-<3 months	3.2% (2.5%, 7.6%)	3.9% (2.1%, 5.6%)	1.4% (1.1%, 2.6%)	1.1% (0.8%, 2.2%)	2.3% (1.6%, 3.8%)	3.0% (2.5%, 5.2%)

3-6 months	1.9% (1.0%, 4.1%)	3.9% (1.7%, 7.5%)	8.2%	0.9% (0.5%, 1.3%)	2.3% (1.7%, 3.1%)	2.0% (0.4%, 5.1%)
**Median % Time <3.0 mmol/L (IQR)**						
0-<3 months	0.5% (0.3%, 2.1%)	0.9% (0.2%, 1.6%)	0.3% (0.0%, 0.5%)	0.1% (0.1%, 0.3%)	0.4% (0.3%, 0.9%)	0.6% (0.5%, 1.2%)
3-6 months	0.3% (0.1%, 0.5%)	0.9% (0.2%, 2.0%)	1.7%	0.3% (0.1%, 0.5%)	0.4% (0.3%, 0.8%)	0.5% (0.1%, 0.8%)
**Glucose CV (%)**						
0-<3 months	37% ± 6%	40% ± 5%	37% ± 5%	37% ± 4%	38% ± 5%	42% ± 6%
3-6 months	39% ± 5%	40% ± 6%	39%	35% ± 0%	39% ± 4%	39% ± 7%
**Glucose SD (mg/dL)**						
0-<3 months	49 ± 12	62 ± 15	55 ± 16	73 ± 7	60 ± 19	77 ± 20
3-6 months	60 ± 14	66 ± 18	47	72 ± 7	62 ± 11	77 ± 18
**Glucose SD (mmol/L)**						
0-<3 months	2.7 ± 0.6	3.4 ± 0.8	3.1 ± 0.9	4.1 ± 0.4	3.3 ± 1.0	4.3 ± 1.1
3-6 months	3.3 ± 0.8	3.7 ± 1.0	2.6	4.0 ± 0.4	3.4 ± 0.6	4.3 ± 1.0

* Plus-minus values are means ± SD. IQR denotes interquartile range.

Supplementary Table 8:
Participant Feedback – Standard care (standard insulin delivery with CGM) participants

Themes / Subthemes	Participant quotations
**Difficulties experienced during the postpartum period**	**1. Managing diabetes in the postpartum period**<br>"For the six months following birth my diabetes control has been difficult. Having 2 children makes for a busy life & fitting in controlling diabetes takes a lot of time and effort" (SC5)<br><br>**2. Labile glucose levels**<br>"My body was and still is adjusting, it has not been the same since pregnancy and birth and my hypo awareness has definitely been left affected. I seem to drop very quick and also spike after meals lots more than before I had my baby" (SC4)<br><br>**3. Hypoglycaemia**<br>"I definitely struggled with hypos a bit in the first few weeks and this made it difficult to look after my baby and also my older child." (SC9)<br><br>**4. Worry about glycaemia and glucose control**<br>"I've been really down about how drastically my glucose control changed from pregnancy to post-natal. My glucose control during pregnancy was probably the best it had ever been, then since giving birth it's been all over the shop with the new (and huge) lifestyle changes, irregular eating patterns and breastfeeding." (SC11)<br><br>**5. Breastfeeding**<br>"I have definitely found it stressful trying to manage my sugars whilst breastfeeding and to some extent knowing my blood glucose the whole time may have actually added to this stress!!" (SC9)
**Benefits of CGM**	**1. Less worry, more confidence**<br>"Still felt a massive relief knowing I had my dexcom on. Having baby And looking after baby is busiest and most tired I've ever been. I was alerted of hypos that I didnt even feel. Same as during my Pregnancy. Which could have saved my life when you think about it. All those times I could have gone too low but didnt." (SC3)<br><br>**2. Changed relationship with diabetes**<br>"I have been on many different treatments & struggled with the management of my condition. I can safely say being able to use the Dexcom completely changed my mental relationship with diabetes as it made it so much easier to manage." (SC5)<br><br>**3. Alleviating difficulties related to managing diabetes in the postpartum period**<br>"It made managing sugars much easier, especially when I was about to feed (was easy to quickly check sugars when my baby was crying to check I was safe to feed and wouldn't hypo. It has allowed me to eat more with less stress. It has also helped build my confidence when out alone with my baby." (SC10)

**Factors that facilitated diabetes self-management and increased feeling of safety**	**1. Alarms**<br>"Alarms that are predictive with Dexcom are helpful and makes me feel safer with [my baby] when my partner is at work. When you're relying on symptoms is really hard because when I'm tired or exhausted or hungry but the alarms really help with that." (SC17)<br><br>**2. Being able to monitor more closely**<br>"Dexcom incredibly helpful to monitor trends and act accordingly with insulin changes." (SC2)<br><br>**3. Being able to share glucose data with other people**<br>"Having this linked up to my husband and parents means it does make this easier as it is always a worry when looking after young children." (SC4)<br><br>**4. Data accessible to healthcare team**<br>"Helped me keep an eye and also helped the new team look at them after having a seizure" (SC7)<br><br>**5. Not having to test / scan**<br>"Easy checking has taken less time so therefore more incline to check in turn keeping me safer and also my baby while in my care." (SC20)<br><br>**6. Accuracy**<br>"Compared to Libre... The only time Dexcom G6 wasn't accurate is when I fell asleep on my arm." (SC17)
**Issues with CGM**	**Device issues**<br>"I have also been quite unlucky with quite a few dodgy sensors so there have been times when I have been reading high or low when it has actually been fine" (SC9)<br><br>**Skin reactions**<br>"I found I was having some severe reactions to the Dexcom adhesive. (This started happening [during pregnancy] however progressively got much worse once [my baby] was born) therefore I required multiple breaks in my Dexcom journey. This was frustrating as I feel like I my diabetes control is much improved when able to use the Dexcom. I spoke to a nurse who works with Dexcom and was offered some different options to try with regards to the reactions however it took 6+ months to find a solution that makes wearing them much more comfortable." (SC5)

Supplementary Table 9:
Participant Feedback - Intervention (HCL) participants

Themes / Subthemes	Participant quotations
**Benefits of HCL**	**1. Better glucose levels**<br>“Amazing control with closed loop system. So pleased with my blood glucose levels the past 6 months. The closed loop system is fantastic & has eased my life significantly” (HCL19)<br><br>**2. Less worry, more confidence and less mental burden**<br>“Made me feel more secure and relaxed knowing that I am less likely to have a hypo. I also feel like it takes less of my mental energy to manage my blood sugars” (HCL7)<br><br>**3. Could focus less on diabetes**<br>“Breastfeeding and sleepless nights were much easier to manage while on closed loop system. I had no concerns about my BG and was able to focus on my recovery and caring for a newborn” (HCL18)<br><br>**4. Better sleep**<br>“I sleep much easier knowing that the closed loop system is looking after me while I sleep.” (HCL14)<br><br>**5. Other unexpected benefits**<br>“My recovery from my c-section was so much smoother and easier than I ever anticipated it to be and I believe this is due to my great blood sugar control and low HBA1C. I believe if I wasn't using the loop I would not be in such good health and that would have made recovery and early motherhood much harder.” (HCL13)
**Factors that facilitated diabetes self-management and increased feeling of safety**	**1. Alarms**<br>“I did sometimes stop feeding baby to treat a hypo whereas last time with no alarms I did think I maybe feel hypo but I'll sort it when she's finished so that felt much safer as it would keep alarming” (HCL4)<br><br>**2. Allowing the HCL to manage glucose levels independently**<br>“I've not had to think much about my blood sugars in these early weeks as I know I can rely on the loop to take care of me. Having that mental headspace and freedom to not be thinking about my blood sugars all the time has allowed me to focus on my child and the value of that can't be underestimated” (HCL13)<br><br>**3. Reduced hypoglycaemia**<br>“I'm much more confident looking after my baby as I know that hypos and other complications have been drastically reduced thanks to this system.” (HCL14)<br><br>**4. Being able to share diabetes management with others**<br>“I can be led by my baby rather than led my diabetes not having to inject at the same time and always be looking for my meter. Helps plan busy times with ease off. Now with the signal sounds triggers a response with my husband and my five year old - if it goes for a low and my son will get a snack. My husband is able to do more to help me - to help deliver boluses if he's cooking and carb counts.” (HCL12)

**Issues with HCL**	**Device issues**<br>"some issues with connection between pump and phone caused some unpredictable glucose levels. resolved with new pump." (HCL18)<br><br>**Second phone**<br>"Some things easy to forget with a new baby e.g taking a second phone everywhere with you." (HCL8)
